# Lower birth weight-for-age and length-for-age z-scores in infants with in-utero HIV and ART exposure: a prospective study in Cape Town, South Africa

**DOI:** 10.1186/s12884-021-03836-z

**Published:** 2021-05-04

**Authors:** Dorothy C. Nyemba, Emma Kalk, Hlengiwe P. Madlala, Thokozile R. Malaba, Amy L. Slogrove, Mary-Ann Davies, Andrew Boulle, Landon Myer, Kathleen M. Powis

**Affiliations:** 1 Division of Epidemiology & Biostatistics, School of Public Health and Family Medicine, University of Cape Town, Anzio Road, Observatory, Cape Town, 7925 South Africa; 2 Centre for Infectious Disease Epidemiology and Research, School of Public Health and Family Medicine, University of Cape Town, Cape Town, South Africa; 3 Department of Paediatrics & Child Health, Faculty of Medicine & Health Sciences, Stellenbosch University, Worcester, South Africa; 4 Ukwanda Centre for Rural Health, Faculty of Medicine & Health Sciences, Stellenbosch University, Worcester, South Africa; 5 Western Cape Government: Health, Cape Town, South Africa; 6 Department of Internal Medicine and Pediatrics, Massachusetts General Hospital, Boston, MA USA; 7 Department of Immunology and Infectious Diseases, Harvard T.H. Chan School of Public Health, Boston, MA USA; 8 Botswana Harvard AIDS Institute Partnership, Gaborone, Botswana

**Keywords:** HIV-exposed uninfected, HIV-unexposed uninfected, Antiretroviral therapy, Weight-for-age, Length-for-age

## Abstract

**Background:**

Successful scale-up of antiretroviral therapy (ART) during pregnancy has minimized infant HIV acquisition, and over 1 million infants are born HIV-exposed but uninfected (HEU), with an increasing proportion also exposed in utero to maternal ART. While benefits of ART in pregnancy outweigh risks, some studies have reported associations between in utero ART exposure and impaired fetal growth, highlighting the need to identify the safest ART regimens for use in pregnancy.

**Methods:**

We compared birth anthropometrics of infants who were HEU with those HIV-unexposed (HU) in Cape Town, South Africa. Pregnant women had gestational age assessed by ultrasound at enrolment. Women living with HIV were on ART (predominately tenofovir-emtricitabine-efavirenz) either prior to conception or initiated during pregnancy. Birth weights and lengths were converted to weight-for-age (WAZ) and length-for-age (LAZ) z-scores using Intergrowth-21st software. Linear regression was used to compare mean z-scores adjusting for maternal and pregnancy characteristics.

**Results:**

Among 888 infants, 49% (*n* = 431) were HEU and 51% (*n* = 457) HU. Of 431 HEU infants, 62% (*n* = 268) were exposed to HIV and antiretrovirals (ARVs) from conception and 38% (*n* = 163) were exposed to ARVs during gestation but after conception (median fetal ARV exposure of 21 weeks [IQR; 17–26]). In univariable analysis, infants who were HEU had lower mean WAZ compared with HU [β = − 0.15 (95% Confidence Interval (CI): − 0.28, − 0.020)]. After adjustment for maternal age, gravidity, alcohol use, marital and employment status the effect remained [adjusted β − 0.14 (95%CI: − 0.28, − 0.01]. Similar differences were noted for mean LAZ in univariable [β − 0.20 (95%CI: − 0.42, − 0.01] but not multivariable analyses [adjusted β − 0.18 (95%CI: − 0.41, + 0.04] after adjusting for the same variables. Mean WAZ and LAZ did not vary by in utero ARV exposure duration among infants who were HEU.

**Conclusion:**

In a cohort with high prevalence of ART exposure in pregnancy, infants who were HEU had lower birth WAZ compared with those HU. Studies designed to identify the mechanisms and clinical significance of these disparities, and to establish the safest ART for use in pregnancy are urgently needed.

**Supplementary Information:**

The online version contains supplementary material available at 10.1186/s12884-021-03836-z.

## Background

Over 1 million women living with HIV (WLHIV) give birth annually [1]. Successful scale-up of maternal antiretroviral treatment (ART) use in pregnancy has dramatically reduced infant HIV acquisition but has resulted in a large and expanding population of infants born HIV-uninfected despite in utero exposure to HIV (HIV exposed uninfected [HEU]) [2]. Several studies have shown that infants who are HEU experience poorer growth, health and survival outcomes compared with their counterparts, infants who are HIV-unexposed (HU) [2–7]. Some studies have shown that fetal growth, measured by an infant’s birth weight and length may be affected by in utero antiretroviral (ARV) exposure [6, 8–10]. Many studies demonstrated that in utero fetal exposure to HIV and ARVs is associated with adverse birth outcomes such as preterm birth, low birthweight and small-for gestational age [11–15]. As the HIV epidemic has matured, the type of ART recommended for use in pregnancy, as well as the proportion of women on ART prior to conception has changed. Identifying the safest ART regimens for use in pregnancy that optimize maternal and child outcomes represents a key public health challenge. Locations with generalized HIV epidemics and high disease burden may be best positioned to provide answers.

South Africa’s antenatal HIV prevalence is one of the highest globally, reported at approximately 30% in 2017 [16]. In this high prevalence setting, more than 95% of pregnant WLHIV receive ART in pregnancy, resulting in the majority of infants who are HEU having exposure to both the HIV virus and ARVs. It is difficult to disentangle the extent to which observed disparities fetal growth or birth outcomes reflect consequences of fetal exposure to HIV or ARVs [2, 17]. However, it is clear that to study adverse events associated with exposure to HIV and ARVs separately, requires separate distinct methodological approaches. To evaluate the effect of HIV exposure specifically, a comparator group is needed of similarly situated mother-infant pairs where women are not living with HIV or receiving any ARVs in pregnancy. To evaluate the effect of ARV exposure specifically, timing and duration of fetal exposure must be studied among infants born to WLHIV. We used prospectively collected data from the B Positive cohort study of infants who are HEU and HU to evaluate associations between in utero exposure to HIV/ART and infant birth anthropometrics, controlling for socio-economic differences. The primary aim of the parent study, the B Positive study, was to monitor the effectiveness, impact and risks of the World Health Organization’s (WHO’s) Option B+ prevention of mother-to-child transmission (PMTCT) of HIV strategy. In this secondary current analysis of B Positive study data, the aim was to evaluate associations between in utero exposure to HIV/ART and infant birth weight-for-age and length-for-age z-scores, as well as birth outcomes of preterm birth, low birthweight and small for gestational age.

## Methods

### Study setting

The B Positive study was a prospective observational study conducted at a large primary healthcare facility in Gugulethu, a peri urban township in Cape Town, South Africa. The facility serves a population of about 350,000 with an estimated antenatal HIV prevalence of 30% [18].

### Study design and study participants

Consecutive pregnant women > 18 years of age were recruited into the study at their first antenatal care (ANC) visit, regardless of HIV status. Study enrolment occurred between January 2017 to July 2018. Women were eligible for this study if they planned to reside in Cape Town with their infants and had a confirmed maternal HIV status at time of study enrolment. For women not known to be living with HIV, a rapid antibody test was used to confirm their HIV status which is standard of care in routine ANC.

### Study procedure

All eligible pregnant women who were able to provide informed written consent were enrolled. Women were followed antenatally for one to three study visits depending on the gestational age (GA) at enrolment. GA was assessed by a dedicated study ultra-sonographer and repeated at all subsequent antenatal visits. Mother-infant pairs were evaluated postnatally at 7 days and birth anthropometrics were abstracted as recorded at birth in South Africa’s child Road to Health Booklet (RTHB). Birth weight of new-borns was measured within 24 h of birth by health facility nurses. To be included in this secondary analysis, a woman had to deliver liveborn, singleton infant and birth weight and/or length data had to be available on the child’s RTHB.

### Data collection

Data collected included maternal demographics, pregnancy history and healthcare information. Questionnaires were administered to all women by trained study interviewers. Pregnant women with a negative HIV test at enrolment based on routine rapid antibody test, had repeat HIV testing up to once every 3 months during the antenatal period and immediately after delivery, as per South Africa’s ANC standard guidelines [19]. For the study, maternal HIV status and testing results were self-reported and confirmed through medical chart review. Per study protocol, all infants were weighed by study staff after removal of clothing and diapers at the 7-days postnatal study visit. Two measurements of infant weight and length were taken at each visit by study staff. Additional medical information was abstracted from antenatal, obstetric, medical and laboratory records including birth weight and length. Study data were collected and managed using Research Electronic Data Capture (REDCap) which is a secure, web-based application designed to support data capture for research studies [20]. REDCap tool is hosted at the University of Cape Town.

### Exposures and outcomes

For this analysis, we used data collected during the antenatal study visits, at birth and 7 days after delivery/birth. We evaluated two primary exposures of interest. The first was exposure to HIV in utero (HEU versus HU), while the second focused on timing of ARV exposure only for infants who were HEU and consisted of a dichotomous variable of either ARV exposure from before conception and during gestation; or after conception but during gestation. Birth weight and length abstracted from the RTHB were used to derive anthropometric primary outcomes, birth weight-for-age (WAZ) and length-for-age (LAZ) z-scores, representing surrogate measures of fetal growth. WAZ and LAZ scores were generated using Intergrowth-21st software, which adjust for infant GA at birth and infant sex [21]. Infant GA at delivery was derived using the GA ascertained from a maternal ultrasound done at enrolment, the date of enrolment and the infant’s date of delivery. Potential confounders identified for this analysis included maternal age, marital status, gravidity and employment status (categorized as formal or informal employment, attending school/college or unemployed). Alcohol use information was collected using a published tool called AUDIT [22], and the variable was a dichotomous variable (yes or no) for any alcohol intake during pregnancy.

### Statistical analysis

Data were analysed using Stata 14.0 (Stata Corporation, College Station, TX, USA) [23]. Maternal and infant characteristics were compared using Wilcoxon test, χ^2^ test or Fisher exact test as appropriate. Proportions of secondary outcomes, preterm birth, low birthweight and small-for-gestational age were compared by in utero HIV exposure (HEU vs HU) and by timing of in utero ARV exposure in HEU infants (before conception vs after conception but during gestation). Univariable and multivariable linear regression models were used to compare the primary outcomes, mean WAZ and LAZ scores first by in utero HIV exposure status, then by timing of in utero ARV exposure in infants who were HEU. All covariates in univariable analyses with a *p*-value of 0.10 were included in multivariable analyses. Additionally, an a priori decision was made to include maternal age in the multivariable model, regardless of univariable *p*-value.

### Ethical considerations

This study was approved by the University of Cape Town’s Faculty of Health Sciences Research Ethics Committee (UCT-HREC) and all women participating in the study provided informed written consent for their own participation and that of their infant’s.

## Results

**Fig. 1:**
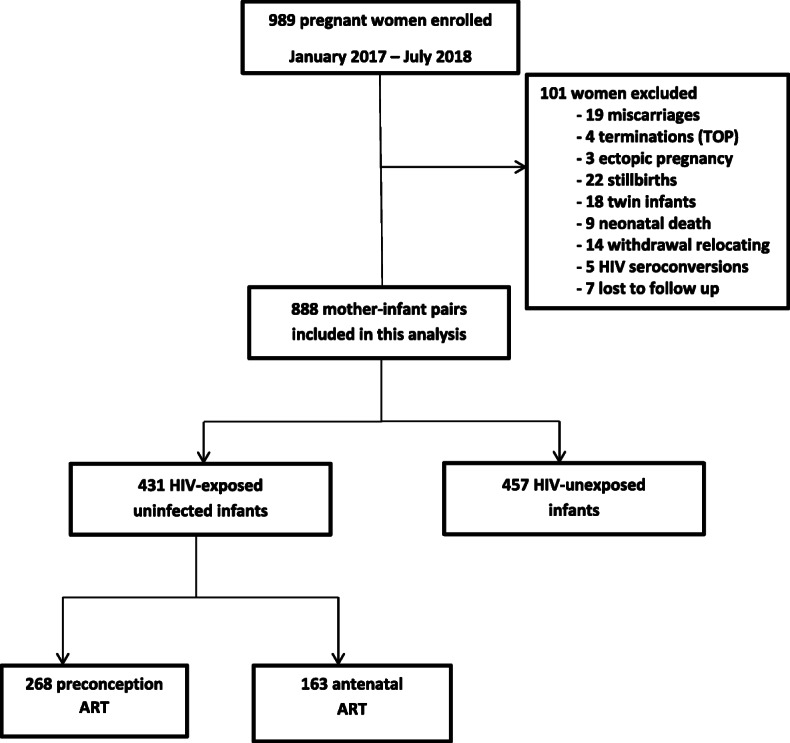
Study flow diagram of participants

Out of 989 women enrolled in the B Positive cohort, 888 mother-infant pairs with live singleton births with birth anthropometry data available were included in this analysis (Fig. 1). Of the 888 infants, 431 (49%) were HEU, while 457 (51%) were HU. For maternal characteristics, WLHIV were older [31 years; IQR 26–35] than women living without HIV [27 years; IQR 23–32 (*p* < 0.001)]; more likely to be multigravid (3 pregnancies; [IQR 2–3] versus 2 pregnancies; [IQR 2–3 (*p* < 0.001)]) and formally employed (37% vs 31%; *p* < 0.001). (Table 1). Of the 431 HEU infants, 268 (62%) were exposed to both HIV and ARV from conception with median gestation of 39 weeks (IQR 38–40), while the remaining 163 (38%) were exposed to ARV after conception but during gestation with median duration of fetal ART exposure of 21 weeks (IQR 17–26). Women who were already on ART at conception were older ([32 years; IQR 29–36] versus [28 years; IQR 25–33 (*p* < 0.001)]) and more likely to be multigravid [3 pregnancies; IQR 2–4] than women who initiated ART during pregnancy [2 pregnancies; IQR 2–3 (*p* < 0.001)]. There were no differences between WLHIV and women without HIV with respect to gestational age at delivery, parity, maternal education, marital status and alcohol use during pregnancy.

**Table 1:** Characteristics of women and new-borns by infant HIV exposure and infant ART exposure

	Women and infants by HIV exposure status (***N*** = 888)				WLHIV and HEU infants by ART exposure status (***N*** = 431)		
	All women 888	HU 457 (51)	HEU 431 (49)	*p*-value *	Preconception ART 268 (62)	Antenatal ART 163 (38)	*p*-value **
**Maternal characteristics**							
Age (years), median (IQR)	**29 (25–33)**	27 (23–32)	31 (26–35)	< 0.001	32 (29–36)	28 (25–33)	< 0.001
GA at enrolment (weeks) median, (IQR)	**19 (14–24)**	20 (14–25)	19 (13–23)	0.05	19 (13–24)	17 (13–22)	0.01
GA at delivery (weeks), median (IQR)	**39 (38–40)**	39 (38–40)	39 (38–40)	0.11	39 (38–40)	39 (38–40)	0.80
ART during (median, IQR)	**–**	–	37 (24–39)		39 (38–40)	21 (17–26)	
**ART initiation**							
Preconception ART	268 (62) *	–	268 (62)				
Antenatal ART	163 (38) *	–	163 (38)				
**Gravidity**							
Median (IQR)	**3 (2–3)**	2 (1–3)	3 (2–3)	< 0.001	3 (2–4)	2 (2–3)	< 0.001
**Parity**							
Median (IQR)	**2 (1–2)**	2 (1–2)	2 (1–2)	0.10	2 (1–2)	1 (1–2)	< 0.001
**ANC visits in this pregnancy**							
1	**191 (22)**	108 (24)	83 (19)	0.27	50 (19)	33 (20)	0.61
2	**171 (19)**	84 (19)	87 (20)		58 (21)	29 (18)	
3	**526 (59)**	265 (58)	261 (61)		160 (60)	101 (62)	
**Education level completed**							
Primary	**43 (5)**	16 (3)	27 (6)	0.03	20 (7)	7 (4)	0.25
Secondary	**827 (93)**	428 (94)	399 (93)		246 (92)	153 (94)	
Tertiary (University)	**18 (2)**	13 (3)	5 (1)		2 (1)	3 (2)	
**Relationship with father of child**							
Married/Cohabiting	**392 (44)**	199 (44)	193 (45)	0.71	133 (50)	60 (37)	0.01
Not married/ Non-cohabiting	**496 (56)**	258 (56)	238 (55)		135 (50)	103 (63)	
**Employment status**							
Formal employment	**301 (34)**	141 (31)	160 (37)	0.001	101 (38)	59 (36)	0.73
Informal employment	**4 (1)**	3 (1)	1 (1)		1 (1)	0 (0)	
Attending school/college	**67 (8)**	50 (11)	17 (4)		9 (3)	8 (5)	
Unemployed	**516 (58)**	263 (57)	253 (58)		157 (59)	96 (59)	
**Alcohol use in pregnancy**							
Yes	**87 (10)**	44 (10)	43 (10)	0.86	25 (9)	18 (11)	0.56
No	**801 (90)**	413 (90)	388 (90)		243 (90)	145 (89)	
**New-born characteristics**							
**Infant Sex**							
Male	**431 (49)**	228 (50)	203 (47)	0.4	131 (49)	72 (44)	0.34
Female	**457 (51)**	229 (50)	228 (53)		137 (51)	91 (56)	
**Preterm delivery**	**101 (11)**	47 (10)	48 (13)	0.29	32 (12)	22 (13)	0.63
**Birth weight (median, IQR)**							
Male	**3.2 (2.8–3.5)**	3.2 (2.9–3.5)	3.1 (2.8–3.4)	0.18	3.2 (2.8–3.4)	3.1 (2.8–3.4)	0.43
Female	**3.1 (2.8–3.4)**	3.2 (2.9–3.4)	3.1 (2.7–3.3)	0.004	3.1 (2.8–3.4)	3.1 (2.6–3.3)	0.58
**Birth length (median, IQR)**							
Male	**50 (48–52)**	50 (49–52)	50 (48–52)	0.21	50 (48–52)	50 (48–52)	0.93
Female	**50 (48–52)**	50 (48–52)	50 (48–51)	0.01	50 (48–52)	49 (47–51)	0.16
**Low birthweight < 2500 g**	**95 (11)**	45 (10)	50 (12)	0.39	31 (11)	19 (12)	0.97
**Small for GA < 10th centile**	**98 (11)**	50 (11)	48 (11)	0.92	32 (12)	16 (10)	0.40
**Birth z-score, mean (SD)**							
**Weight-for-age**	**888**	0.08 (1.05)	− 0.07 (0.99)	0.03	−0.05 (1.02)	− 0.10 (0.94)	0.65
**Length-for-age**	**864**	0.82 (1.70)	0.61 (1.58)	0.07	0.65 (1.626)	0.55 (1.53)	0.53

*IQR* Interquartile range, *SD* Standard deviation, *n* Number of participants, *GA* Gestational age, *ANC* Antenatal clinic

*HEU* HIV-exposed uninfected, *HU* HIV-unexposed, *ART* Antiretroviral therapy

* *P*-value from chi-square or Fisher’s exact test, comparison between HU and HEU

**P-value from chi-square or Fisher’s exact test, comparison in HEU between preconception ART and antenatal ART

Infants who were HEU (both male and female) had lower birth weight, [3.1 kg; IQR 2.8–3.4] and [3.1 kg; IQR 2.7–3.3] compared to infants who were HU, [3.2 kg; IQR 2.9–3.5] and [3.2 kg; IQR 2.9–3.4] (*p* = 18 and *p* = 0.004) respectively (Table 1). In univariable analysis (Table 2), mean WAZ was lower among infants who were HEU compared with infants who were HU [β = − 0.15 (95% Confidence Interval (CI): − 0.28, − 0.02), *p* = 0.02]. After adjusting for maternal age, gravidity, alcohol use, marital and employment status, mean WAZ at birth remained significantly lower for infants who were HEU compared to those who were HU [adjusted β − 0.14 (95%CI: − 0.28, − 0.01), *p* = 0.04]. Similar differences were noted for mean LAZ comparison with univariable linear regression analysis [β − 0.20 (95%CI: − 0.42, − 0.02), *p* = 0.04]. However, in the multivariable analysis, there was no significant mean LAZ difference [β − 0.18 (95%CI: − 0.41, + 0.04), *p* = 0.11] after adjusting for the same variables.

**Table 2:** Univariable and Multivariable Linear regression for comparison of WAZ and LAZ between HEU and HU infants

Anthropometric measure			Univariable		Multivariable	
	Predictor	N	β (95% CI)	*P*-value	β (95% CI)	*P*-value
Weight for age Z-score (WAZ)	HU	457	Ref		Ref	
	HEU	431	−0.15 (−0.28; − 0.02)	0.02	− 0.14 (− 0.28; − 0.01)	0.04
Length for age Z-score (LAZ)	HU	420	Ref			
	HEU	400	−0.20 (− 0.42; − 0.01)	0.04	− 0.18 (− 0.41; + 0.04)	0.11

Adjusted for maternal age, gravidity, alcohol use, marital and employment status,

*HEU* HIV-exposed uninfected, *HU* HIV-unexposed

β: mean change in z-score between HEU and HU, *CI* Confidence interval, *N* Number of participants

When we restricted the population to HEU infants only (Table 3), WAZ and LAZ for HEU infants exposed to ARVs after conception were not different to infants exposed to ARVs from conception.

**Table 3:** Univariable and Multivariable Linear regression for Comparison of WAZ and LAZ between in utero preconception ARVs exposure and antenatal ARVs exposure

Anthropometric measure			Univariable		Multivariable	
	Predictor	N	β (95% CI)	*P*-value	β (95% CI)	*P*-value
Weight for age Z-score (WAZ)	From conception ARVs	268	Ref		Ref	
	Antenatal ARVs	163	−0.04 (− 0.23; + 0.15)	0.65	− 0.03 (− 0.23; + 0.17)	0.77
Length for age Z-score (LAZ)	From conception ARVs	262	Ref			
	Antenatal ARVs	158	−0.10 (− 0.41; + 0.21)	0.53	− 0.08 (− 0.41; + 0.24)	0.62

Adjusted for maternal age, gravidity, alcohol use, marital and employment status

*HEU* HIV-exposed uninfected, *CI* Confidence interval, *N* Number of participants

β: mean change in z-score in between from conception ARVs exposure and antenatal (after conception but during pregnancy) ARVs exposure

There were no significant differences between WLHIV and women without HIV with respect to gestational age at delivery, parity, maternal education, marital status and alcohol use during pregnancy. Preterm birth, low birthweight and small-for-gestational age were similar among WLHIV compared to women living without HIV.

## Discussion

In this prospective cohort of pregnant women seeking ANC at a public health care facility in a peri-urban township in South Africa, we found that infants who were HEU experienced lower mean WAZ and LAZ at birth compared to infants who were HU (Tables 1 and 2). Furthermore, among the infants who were HEU, mean WAZ and LAZ did not vary by timing of ARV exposure, either from before conception or initiated later in gestation.

Our finding of lower WAZ at birth among infants who were HEU is consistent with several studies in African populations [2, 3, 6, 8]. While some studies were conducted prior to universal maternal ART, others are similar to our cohort with lower WAZ noted in WLHIV who receive ART. Similarly, LAZ was lower in infants who were HEU, but the difference was attenuated after adjusting for measured confounders. Our LAZ findings approximate that from other studies done in African populations [2, 3, 6]. The lack of a significant difference could be due to limited statistical power to detect a larger effect. Our study is in the era of universal ART in pregnancy and uniquely includes a group of women who initiated ART prior to conception. Although there are overwhelming benefits of universal ART for both the mother and the infant, it is critical that short- and long-term potential risks be systematically studied using sound methodological approaches, so that the safest regimens for use in pregnancy are identified. The B Positive study presents an optimal study design, as mother-infant pairs were enrolled from the same community with similar socio-behavioural and socio-economic conditions. In our analysis, we included only liveborn singleton infants. This excludes stillbirths who often have intrauterine growth restrictions as a result of placenta insufficiency potential due to HIV and or ARVs [10], and neonatal deaths. It is plausible that higher maternal HIV viral load could influence placental development during pregnancy. A study from Botswana found that some ART regimens may lead to placental insufficiency due to impaired endothelial function [10]. A forthcoming study on pregnancy and birth outcomes is critical to identify the effect of in utero fetal exposure to ARVs.

When we restricted our analysis to infants who were HEU, focusing on in utero fetal exposure to ART, there was no difference in anthropometric birth measures by timing of an infant’s exposure, either prior to conception or sometime after conception but during gestation. This is consistent with findings from a study of infants who were HEU in Brazil [24]. Unfortunately, a stratified analysis by ARV drug class was not practical, as most of the women in our study sample (80%), were on a first line efavirenz-based (EFV) regimen. Studies that can be stratified by ARV drug class are urgently needed to establish the safest ARV drugs for use in pregnancy and breastfeeding period [25, 26]. In analyzing anthropometrics of infants who were HEU, we were unpowered to evaluate outcomes by gestational duration of ARV exposure, and therefore derived a dichotomous variable. However, understanding if there is a “dose response” between in utero ARV exposure and birth anthropometrics also represent a priority research area.

Our study had several strengths. The study had a comparator group of infants who were HU from the same community, with common socio-behavioural and economic characteristics between infants who were HEU and those who were HU. Another strength was use of a robust gestational age estimates by antenatal ultrasound. We used Intergrowth-21st New-born Standards to generate WAZ and LAZ which adjusts for gestational age and infant sex [21]. In the sub-group of infants who were HEU, we had information on timing of ARV exposure. Our study also has some limitations. Single site data from a peri-urban South Africa community might not be generalizable to other settings like rural areas with different backgrounds. Secondly, we were unable to demonstrate causal effects due to potential unmeasured confounders, always a concern in observational research. However, the study and analytic approach were designed to minimize confounding.

## Conclusion

Despite universal ART treatment during pregnancy and breastfeeding, which has improved maternal health and significantly reduced infant HIV acquisition, fetal growth remains impaired for infants with exposed to in utero HIV/ART compared to infants born to mothers without HIV. Studies to identify clinical significance of growth disparities between HEU infants and HU infants are urgently needed as well as establishing the safest ARV drug for use in pregnancy.

## Supplementary Information

**Additional file 1.**

**Additional file 2.**

**Additional file 3.**

**Additional file 4.**

## Data Availability

The datasets used and analysed during this current study are available from corresponding author on request.

## Abbreviations

ANC: Antenatal clinic
ARV: Antiretroviral
ART: Antiretroviral therapy
GA: Gestational age
HEU: HIV-exposed uninfected
HU: HIV-unexposed
IQR: Interquartile range
LAZ: Length-for-age z-score
PMTCT: Prevention of mother-to-child transmission
WAZ: Weight-for-age z-score
WLHIV: Women living with HIV
SD: standard deviation
