# Supplementary material for: Lower birth weight-for-age and length-for-age z-scores in infants with in-utero HIV and ART exposure: a prospective study in Cape Town, South Africa
Source: BMC Pregnancy Childbirth. 2021 May 4;21:354. doi: 10.1186/s12884-021-03836-z (PMC8097797; doi:10.1186/s12884-021-03836-z)
Supplement: Supplementary file 1 — Additional file 1. [file 12884_2021_3836_MOESM1_ESM.pdf]

PWID: _ _ _ _ _ _ - _

MATERNAL DEMOGRAPHICS & CLINICAL HISTORY

This CRF applies to ALL enrolled BPOS participants

Visit Date								
D	D	M	M	M	Y	Y	Y	Y

Visit Code	
**A**	**1**

A: SOCIODEMOGRAPHIC INFORMATION

**1.** What is your date of birth?	______ /______ /________<br>DD MMM YYYY
**2.** What language do you speak at home?	☐ isiXhosa<br>☐ isiZulu<br>☐ Afrikaans<br>☐ English<br>☐ Other, *specify:* ____________________
3. What is the highest level of schooling or education that you have completed?	☐ Grade: ______<br>**OR**<br>☐ Standard: ______<br>☐ Postsecondary, specify ________________<br>☐ None
**4.** Are you currently working or studying?	☐ Yes<br>☐ No → ***SKIP to Q8***
5. If yes, which one of the following best describes what you do?<br><br>Choose **ONE** only	☐ Employed full-time<br>☐ Employed part-time<br>☐ Informal job/hawker<br>☐ Attending school/learner<br>☐ Attending tertiary education (University/College)
6. Do you earn an income?	☐ Yes<br>☐ No → ***SKIP to Q8***

Initials of interviewer: ____

**7.** How much income do you earn per month?	☐ Less than R1 000 per month<br>☐ 1 000 to R5 000 per month<br>☐ R5 000 to R10 000 per month<br>☐ R10 000 to R15 000 per month<br>☐ More than R15 000 per month
**8.** Do you currently receive any social assistance in the form of government grants?	☐ Yes<br>☐ No →***SKIP to Q11***
**9.** Which type of government grant do you receive?	☐ Children's grant<br>☐ Disability grant<br>☐ Care Dependency grant<br>☐ Other, please specify: ___________________________
**10.** How many of each of the grants mentioned above (Q9) do you receive?<br><br>**Please specify number**	☐ Children's grant # of grants __________<br>☐ Disability grant # of grants __________<br>☐ Care Dependency grant # of grants__________<br>☐ Other, specify: ____________ # of grants __________
**11.** What kind of home do you live in?	☐ Shack/informal dwelling<br>☐ Formal house<br>☐ Flat/council home<br>☐ Other Specify: ___________________________

Initials of interviewer: _____

12. Does your house have the following<br><br>**Respond to ALL**	a. *A toilet inside*<br>☐Yes ☐No	b. *Running water inside*<br>☐Yes ☐No
	c. Electricity inside<br>☐Yes ☐No	d. *A refrigerator*<br>☐Yes ☐No
	e. *A landline telephone*<br>☐Yes ☐No	f. *A television*<br>☐Yes ☐No

**13.** Including yourself, how many people (adults and children) live in your house?	Number of people: ___________
**14.** How many times have you been pregnant (including current pregnancy)?	*Number of pregnancies*: ___________
**15.** How many children have you given birth to?	*Number of children:* ☐___________<br>☐ None
**16.** How many of these children are living?	*Number of children:* ☐___________<br>☐ None

B1: TB HISTORY

We are now going to ask you a few questions about your previous medical history including TB and HIV.

**17.** During your current pregnancy, has a doctor or nurse told you that you have TB?	☐ Yes<br>☐ No →***SKIP to Q22***
**18.** When did you receive this TB diagnosis?	______ /_______ /_________<br>DD MMM YYYY
19. Where did you receive this TB diagnosis?	*Name of clinic*: ___________________________
20. Where in your body was the TB (e.g., lungs, other location)?	*Place in body:* _____________________
21. Did you receive treatment for TB?	☐ Yes<br>☐ No

Initials of interviewer: ____

**22.** Other than during this pregnancy has a doctor or nurse ever told you that you have TB?	☐ Yes<br>☐ No → ***SKIP to Q28***
**23.** When did you receive this TB diagnosis from Q22 (not during this pregnancy)?	______ /_______ /________<br>DD MMM YYYY
**24.** Did you receive treatment for TB when last diagnosed (From Q22)?	☐ Yes<br>☐ No
**25.** How many times in total have you been treated for TB?	*Number of times*: ____________
**26.** Where did you receive your TB treatment (From Q22)?	*Name of clinic*: ____________
**27.** How long was your TB treatment the last time you were diagnosed?	☐ 6 months<br>☐ 8 months<br>☐ 9 months<br>☐ On-going<br>☐ Other, specify ____________<br>☐ Don't know
**B2: HIV HISTORY**	
**28.** Have you ever been tested for HIV?	☐ Yes<br>☐ No
**29.** Are you HIV-negative or HIV-positive?	☐ Negative<br>☐ Positive<br>☐ Don't know

Initials of interviewer: _____

NB: ***Please complete HIV INFORMATION for <u>ALL PARTCIPANTS</u>***

Signed Interviewer completing CRF: ______________________________ Date: _______ / __________ / _________
DD MMM YYYY

Signed QC Officer: ______________________ Date: _______ / __________ / _________
DD MMM YYYY

Signed Study Coordinator: ______________________ Date: _______ / __________ / _________
DD MMM YYYY

Initials of interviewer: ____
