# Supplementary material for: Lower birth weight-for-age and length-for-age z-scores in infants with in-utero HIV and ART exposure: a prospective study in Cape Town, South Africa
Source: BMC Pregnancy Childbirth. 2021 May 4;21:354. doi: 10.1186/s12884-021-03836-z (PMC8097797; doi:10.1186/s12884-021-03836-z)
Supplement: Supplementary file 2 — Additional file 2. [file 12884_2021_3836_MOESM2_ESM.pdf]

MATERNAL MALE PARTNER INVOLVEMENT DURING PREGNANCY

To be completed by ALL enrolled participants

Visit Date								
D	D	M	M	M	Y	Y	Y	Y

Visit Code	
**A**	**1**

**A: ROMANTIC RELATIONSHIPS**	
**We are going to ask you some questions about your previous and current romantic relationships.**	
**1.** Were you in a relationship when you got pregnant?	☐ Yes<br>☐ No → **SKIP TO Q3**
**2.** Is the current partner you were with the father of your unborn baby?	☐ Yes<br>☐ No
**3.** Are you currently in a relationship?	☐ Yes<br>☐ No → **SKIP TO Q10**
**4.** How would you describe your current romantic relationship?	☐ Married (including traditional marriage), living together<br>☐ Married, not living together<br>☐ Not married, living together<br>☐ Not married, not living together<br>☐ Other, Specify : ______________________
**5.** How long have you been in a relationship with this current person?	Duration in:<br>Months : _____ **Or** Years: _______

B: YOUR RELATIONSHIP WITH YOUR CURRENT PARTNER	
**We are now going to ask you some questions about your relationship with your current partner.**	
**6.** What is your current partner's date of birth?	______ /_______ /________<br>DD MMM YYYY<br>If date is unknown, please provide age<br>Age: _____
**7.** What is the highest level of education that your current partner has completed?	☐ Grade: : _______<br>**Or**<br>Standard: _______<br>☐ Postsecondary, specify _________<br>☐ None
**8.** Is your current partner employed and/or studying?	☐ Yes<br>☐ No → **SKIP TO Q10**
**9.** Which one of the following best describes what he does?	☐ Employed full-time<br>☐ Employed part-time<br>☐ Informal job/hawker<br>☐ Attending school/learner<br>☐ Attending tertiary education (University/College)
**C: SUPPORT YOUR CURRENT PARTNER PROVIDES DURING YOUR PREGNANCY**	
**We are now going to ask you some questions about the role your PARTNER plays since you became pregnant.**	
**10.** Is your current partner supportive of your pregnancy?	☐ Not supportive at all<br>☐ Slightly supportive<br>☐ Moderately supportive<br>☐ Considerably Supportive<br>☐ Extremely supportive

**11.** How much can you rely on your current partner for help with your pregnancy and your baby?	☐ Not at all<br>☐ Slightly/not very often<br>☐ Moderately/some of the time<br>☐ Considerably/most of the time<br>☐ Extremely/all of the time
**12.** Which of the following does your current partner do**? Please select ALL that apply**	☐ Accompanies me to antenatal visits<br>☐ Knows when my antenatal visits are scheduled for<br>☐ Provides financial support for my antenatal visits<br>☐ Discusses what happens during antenatal visits with me.<br>☐ Other, please specify: ____________________
**13.** Which of the following would you like your partner to do? **Please select ALL that apply.**	☐ To provide financial support for my antenatal visits<br>☐ To know when my antenatal visits are<br>☐ To discuss what happens during antenatal visits with me<br>☐ To accompany me to antenatal visits<br>☐ Other, *please specify*: ____________________
**D: SUPPORT THE FATHER OF THE BABY PROVIDES DURING YOUR PREGNANCY**	
**We are now going to ask you some questions about the role THE FATHER OF THE BABY plays since you became pregnant.**	
**14.** Is the father of the baby supportive of your pregnancy?	☐ Yes<br>☐ No → **STOP HERE!!!!**
**15.** How supportive of your pregnancy is the father of the baby ?	☐ Slightly supportive<br>☐ Moderately supportive<br>☐ Considerably Supportive<br>☐ Extremely supportive

**16.** How much can you rely on the father of the baby for help with your pregnancy?	☐ Not at all<br>☐ Slightly/not very often<br>☐ Moderately/some of the time<br>☐ Considerably/most of the time<br>☐ Extremely/all of the time
**17.** Which of the following does the father of the baby do? **Please select ALL that apply**	☐ Accompanies me to antenatal visits<br>☐ Knows when my antenatal visits are scheduled for<br>☐ Provides financial support for my antenatal visits<br>☐ Discusses what happens during antenatal visits with me<br>☐ Other, *please specify*: ___________________
**18.** Which of the following would you like the father of the baby to do? **Please select ALL that apply**	☐ To provide financial support for my antenatal visits<br>☐ To know when my antenatal visits are<br>☐ To discuss what happens during antenatal visits with me<br>☐ To accompany me to antenatal visits<br>☐ Other, please specify: ___________________

Signed Interviewer completing CRF: ______________________________ Date: _______ / __________ / _________
DD MMM YYYY

Signed QC Officer:______________________ Date: _______ / __________ / _________
DD MMM YYYY

Signed Study Coordinator:______________________ Date: _______ / __________ / _________
DD MMM YYYY
