# Supplementary material for: Lower birth weight-for-age and length-for-age z-scores in infants with in-utero HIV and ART exposure: a prospective study in Cape Town, South Africa
Source: BMC Pregnancy Childbirth. 2021 May 4;21:354. doi: 10.1186/s12884-021-03836-z (PMC8097797; doi:10.1186/s12884-021-03836-z)
Supplement: Supplementary file 3 — Additional file 3. [file 12884_2021_3836_MOESM3_ESM.pdf]

PWID: _ _ _ _ _ _ - _ _

MATERNITY CASE RECORD ABSTRACTION FORM

This form applies to ALL enrolled BPOS participants
Complete after conclusion of routine Antenatal Care Booking Visit Procedures

**Date of Data Abstraction:**	____ / ________ / ______ (DD / MMM / YYYY)	**Initials of Data Abstractor:**	
**Participant Full Name:**	**Name:** **Surname:**	**Participant Date of Birth:**	____ / ________ / ______ (DD / MMM / YYYY)
**Participant National ID**		**Provincial Folder Number:** (specify 3 letter facility prefix; GUP or other)	G U P
**Gravidity**		**Parity**	
**Miscarriages**			

Previous Pregnancies (as per Maternity Chart)

Year	Gestation	Delivery	Weight	Sex	Outcome	Complications

Medical & General History

**THIS Pregnancy** <br>**Healthy:** ☐ YES ☐ NO <br>If NO tick all options that apply	☐ Hypertension ☐ Epilepsy ☐ Cardiac ☐ TB ☐ Other: ____________________ <br>Further details if any of the above selected: ____________________ <br>**Any Medications:** *please specify* ____________________
**Previous Pregnancies** <br>☐ Family History	☐ Twins ☐ Diabetes ☐ TB ☐ Congenital ☐ Other: ____________________ <br>Further details if any of the above selected: ____________________ ____________________

PWID: _ _ _ _ _ _ - _ _

Details of Booking Examination

**Date**	____ / ______ / ______ (DD MMM YYYY)	**BP**	____ / ____ (Systolic Diastolic)	**HB**	____ g/dl
**Height**: ________ cm		**Weight**: ______ kg		**MUAC**: ________ cm	
**SFH**: ________ cm					

**RPR Result**	Neg	Pos	**Titre if pos:** ______	**Rhesus:** ______	**ABO Bloodgroup:** ______

**1st HIV Test**	___ /____ /_____ (DD MMM YYYY)	**RESULT**			**2nd HIV Test**	___ /____ / ____ (DD MMM YYYY)	**RESULT**		
		POS	NEG	Decline			POS	NEG	Decline
**On ART**	Y	N	**Initiation Date**	____ / _____ / _____ (DD MMM YYYY)	**Regimen**				

EDD Estimation

**Date of assessment #1**	___ / _____ / ____	**# 1: Type**	Date / LNMP	SFH	USS
Gestational Age (weeks):		EDD: ____ / ____ / ____ (DD MMM YYYY)			
**Date of assessment #2**	___ / _____ / ____	**#2: Type**	Date / LNMP	SFH	USS
Gestational Age (weeks):		EDD: ____ / ____ / ____ (DD MMM YYYY)			
**Date of assessment #3**	___ / _____ / ____	**#3: Type**	Date / LNMP	SFH	USS
Gestational Age (weeks):		EDD: ____ / ____ / ____ (DD MMM YYYY)			

Additional Notes:

____

____

____

Signed by Abstractor: ____________________
