# Supplementary material for: Lower birth weight-for-age and length-for-age z-scores in infants with in-utero HIV and ART exposure: a prospective study in Cape Town, South Africa
Source: BMC Pregnancy Childbirth. 2021 May 4;21:354. doi: 10.1186/s12884-021-03836-z (PMC8097797; doi:10.1186/s12884-021-03836-z)
Supplement: Supplementary file 4 — Additional file 4. [file 12884_2021_3836_MOESM4_ESM.pdf]

PWID: _ _ _ _ _ - _ _

ODA

OBSTETRICS ABSTRACTION FORM

This form applies to ALL enrolled BPOS Participants
Complete after delivery

**Date of Data Abstraction:**	____ / ____ / ____ (DD MMM YYYY)	**Initials of Data Abstractor:**	
**Participant Full Name:**		**Participant Date of Birth:**	____ / ____ / ____ (DD MMM YYYY)
**Participant National ID**		**Provincial Folder Number:** specify 3 letter facility prefix; GUP or other	G U P
**Source Document**	☐ Maternity Case Record ☐ Delivery Register ☐ PMTCT Register (incl DISA/Trakcare)<br>☐ Other, Please Specify: ____________		

MOTHERS DETAILS

**Name of clinic**			**GA at 1st visit**			**Booking Date**	____ / ____ / ____ (DD MMM YYYY)
**Hb**			**Rhesus**	Pos	Neg	**Syphilis**	Pos / Neg
**HIV results**	Pos	Neg	**HIV retest**	Pos	Neg	**ART**	Y / N
**Regimen**	☐ NR				**ART Start Date**		____ / ____ / ____ (DD MMM YYYY)
**Estimated Gestational Age**	Weeks:________<br>If weeks not recorded:<br>☐ Term ☐ Preterm		**Gestational Age estimated by**			☐ Dates ☐ Palpitation<br>☐ SFH ☐ Ultrasound Scan<br>☐ Not Reported	

LABOUR DETAILS

**Delivery Date**	____ / ____ / ____ (DD MMM YYYY)			**Delivery time**	____ : ____	
**Fetal Heart**	**Present**	**Absent**	**uncertain**	**Fetal Distress**	Y	N / NR
**Place of Delivery**	☐ GMOU ☐ MMH ☐ GSH<br>☐ Other, Specify:<br>____________		**Delivery Method** *Please tick all methods used during this delivery*		☐ NVD ☐ C/S ☐ Induction of labour<br>☐ Vacuum ☐ Forceps ☐ BBA	

**If C/S, Primary indication**	☐ Fetal distress ☐ Obstructed labour<br>☐ Twins/Triplets ☐ Pre-eclampsia /eclampsia<br>☐ APH ☐ Previous C/S<br>☐ Other, specify: ____________	**If by C/S, was it performed after membrane rupture?**	☐ Yes, Duration:_____ Mins<br>☐ No<br>☐ NR

**Please tick all major medical and/or obstetric conditions the mother experienced during pregnancy and/or during delivery.**	☐ Chorio amnionitis<br>☐ IUGR<br>☐ Diabetes mellitus<br>☐ PPH<br>☐ Cervical tear<br>☐ Other, Specify: ____________	☐ Sepsis<br>☐ Hypertension<br>☐ Preterm labour<br>☐ Prolonged ROM<br>☐ Perineal tear	☐ UTI / Pyelonephritis<br>☐ Pre-eclampsia/ eclampsia<br>☐ APH<br>☐ Prolonged labour<br>☐ Episiotomy

INFANT DETAILS

**Infant DOB**	____ / ______ / _____<br>DD MMM YYYY	**Infant DOB for Twin B**	____ / ______ / _____<br>DD MMM YYYY
**Gender**	☐ Male ☐ Female	**Gender Twin B**	☐ Male ☐ Female
**Outcome**	☐ Alive ☐ Stillborn ☐ NND	**Outcome Twin B**	☐ Alive ☐ Stillborn ☐ NND
**Resuscitation**	☐ Yes ☐ No	**Resuscitation Twin B**	☐ Yes ☐ No
**Birthweight**	____________ g	**Birthweight Twin B**	____________ g
**Head circumference**	____________ cm	**Head circumference Twin B**	____________ cm
**Length**	____________ cm	**Length Twin B**	____________ cm
**APGAR Score**	1 min: ______ 5 min: ______	**APGAR Score Twin B**	1 min: ______ 5 min: ______
**Congenital Abnormalities?**	☐ Yes ☐ No ☐ NR<br>Specify:____________	**Congenital Abnormalities Twin B**	☐ Yes ☐ No ☐ NR<br>Specify:____________
**Polio Vaccine?**	☐ Yes ☐ No ☐ NR	**Polio Vaccine Twin B**	☐ Yes ☐ No ☐ NR
**BCG Vaccine?**	☐ Yes ☐ No ☐ NR	**BCG Vaccine Twin B**	☐ Yes ☐ No ☐ NR
**NVP at birth?**	☐ Yes ☐ No ☐ NR	**Twin B NVP at birth?**	☐ Yes ☐ No ☐ NR
**Feeding Option**	☐ Breast ☐ Formula		

DISCHARGE SUMMARY

**Family planning choice**	☐ Oral contraceptive<br>☐ Implant	☐ Injectable<br>☐ Tubal ligation	☐ IUD<br>☐ Other: ____________
**Date of discharge**	______ / ________ / ________<br>DD MMM YYYY		
